# Outbreak of bacteremia caused by *Ralstonia insidiosa* isolated from a contaminated blood gas syringe

**DOI:** 10.55730/1300-0144.5967

**Published:** 2024-12-23

**Authors:** Elif Ayça ŞAHİN, Özge ÖZGEN TOP, Pınar AYSERT YILDIZ, Elif Seren TANRIVERDİ, Hasan Selçuk ÖZGER, Barış OTLU, Özlem GÜZEL TUNÇCAN, Murat DİZBAY, Ayşe KALKANCI, Kayhan ÇAĞLAR

**Affiliations:** 1 Department of Medical Microbiology, Faculty of Medicine, Gazi University, Ankara, Turkiye; 2 Department of Infectious Disease, Faculty of Medicine, Gazi University, Ankara, Turkiye; 3 Department of Medical Microbiology, Faculty of Medicine, Inonu University, Malatya, Turkiye

**Keywords:** *Ralstonia* sp., AR-PCR, blood culture, bacteremia, contamination

## Abstract

**Background/aim:**

*Ralstonia* species are opportunistic, waterborne microorganisms known for their ability to survive and proliferate in a wide range of water-based environments. They can contaminate solutions used for patient care and cause hospital outbreaks due to contaminated solutions. The aim of this study was to investigate the source and clonal relationship of *Ralstonia insidiosa* bacteremia detected in 28 patients between August and December 2021, as part of an unusual outbreak.

**Materials and methods:**

Active prospective surveillance studies were conducted, and environmental samples, including saline, antiseptic, and antibiotic solutions, injectors, arterial blood gas syringes, tap water, and hand soap, were collected from wards to determine the source of the outbreak. An arbitrary-primed polymerase chain reaction (AP-PCR) genotyping method was used to determine the clonal relationship between the isolates.

**Results:**

All the samples were cultured, and *R. insidiosa* was isolated from arterial blood gas syringes with the same location and time-based identifier (LOT number). All the arterial blood gas syringes were recalled from the hospital departments and sent back to the manufacturer. The outbreak was reported to the national health authorities. Clonal analysis between isolates from the patients and the blood gas syringes was performed using AP-PCR. It was observed that the *R. insidiosa* isolates were monoclonal and identical.

**Conclusion:**

It was concluded that these contaminated arterial blood gas syringes caused the *R. insidiosa* bacteremia, especially in immunocompromised patients.

## 1. Introduction

*Ralstonia* species are opportunistic, waterborne organisms that can survive and grow in various water sources, such as tap water, industrial water distribution systems, and laboratory-purified water systems [1]. Therefore, these microorganisms can contaminate solutions used for patient care, such as saline solutions, intravenous drugs, distilled water, or respiratory solutions [2–4]. Contamination of these solutions can cause outbreaks of invasive infections, such as bloodstream infections (BSIs), osteomyelitis, and meningitis [5]. The most commonly detected *Ralstonia* species is *Ralstonia pickettii* [6]. However, *Ralstonia mannitolilytica*, *Ralstonia solanacearum*, and *Ralstonia insidiosa* can also cause human infections. *R. insidiosa*, the bacterium most closely related to *R. pickettii* [7], can cause hospital outbreaks due to contaminated solutions. This study presents an outbreak of bacteremia caused by *R. insidiosa* that was related to heparinized blood gas syringes in our hospital.

## 2. Materials and methods

### 2.1. Study design and settings

The Gazi University Hospital Microbiology Laboratory and the Infection Control Committee noticed an unusual increase in the number of patients with *R. insidiosa* bacteremia between August and December 2021. During this period, *R. insidiosa* was detected in the blood cultures of 28 patients. Active prospective surveillance studies were conducted, and environmental samples, including saline, hand soap, antiseptic and antibiotic solutions, injectors, arterial blood gas syringes, and tap water, were collected from the wards to determine the source of the outbreak. Four patients were selected for clonal analysis to represent different hospital units and investigate the potential transmission dynamics: One patient from the Pediatric Services, representing bacteremia cases in the pediatric population. One patient from the General Internal Medicine Services, chosen to reflect adult patients with diverse medical conditions.

Two patients from the Neonatal Intensive Care Unit (NICU), included due to concerns about vulnerable neonates and possible environmental contamination, as well as to determine if multiple cases in the NICU were clonally related. These patients were chosen to explore possible links between environmental sources and bacteremia across distinct clinical settings. An arbitrary-primed polymerase chain reaction (AP-PCR) genotyping method was used to determine the clonal relationship between the isolates [8].

### 2.2. Microbiological identification of the isolates

Pediatric and adult patients with *R. insidiosa* growth in peripheral and/or catheter blood cultures in the wards and intensive care units (ICU) were included in the study. Blood cultures were incubated in BacT/ALERT 3D instruments (BioMérieux, Craponne, France). The isolates were identified using a matrix-assisted laser desorption/ionization time-of-flight mass spectrometer (Bruker Daltonics, Bremen, Germany). First, 10 mL of environmental samples were inoculated into aerobic blood culture bottles and incubated in BacT/ALERT 3D instruments (BioMérieux). Saline solution was drawn into the injectors and the arterial blood gas syringes were inoculated onto 5% sheep blood agar, chocolate agar, and eosin methylene blue (EMB) agar. The plates were incubated at 35–37 °C and inspected for growth at 24 and 48 h.

### 2.3. Antimicrobial susceptibility testing (disc diffusion test)

A bacterial suspension equivalent to a 0.5 McFarland standard (approximately 1–2 × 10^8 CFU/mL) was prepared using sterile saline or an appropriate broth. Mueller-Hinton agar plates were used for antibiotic susceptibility testing. The prepared bacterial suspension was uniformly swabbed across the surface of the agar using a sterile cotton swab to ensure even distribution. Antibiotic disks were placed on the agar surface with a minimum distance of 24 mm between the centers of the disks to prevent overlapping zones of inhibition. The plates were incubated at 35–37 °C for 16–18 h. Following incubation, the diameters of the inhibition zones around the antibiotic disks were measured in millimeters. The results were interpreted according to the criteria set by the European Committee on Antimicrobial Susceptibility Testing for similar nonfermentative gram-negative bacteria, in the absence of specific breakpoints for *R. insidiosa*.

### 2.4. Clonal relationships analysis

The clonal relationships of the isolates were determined using the AP-PCR genotyping method, as described by Kuzucu et al. [8]. Bacterial DNA was extracted using a QIAamp DNA Mini Kit (Qiagen, GmbH, Hilden, Germany). The extracted DNA samples were stored at −80 °C until use. The AP-PCR was performed with a reaction mixture containing M13 primer (5′-GAG GGT GGC GGT TCT-3′), template DNA, Taq DNA polymerase (Promega Corp., Madison, WI, USA), deoxynucleoside triphosphates (dNTPs), magnesium chloride (MgCl2), and a 10X amplification buffer. Amplification was carried out using a GeneAmp PCR System 9700 (Applied Biosystems, Waltham, MA, USA). Electrophoresis was performed on the amplified products using 2% agarose gel stained with ethidium bromide. The DNA bands were visualized under ultraviolet light using a Kodak Gel Logic 200 Imaging System (Eastman Kodak Co., Rochester, NY, USA). The banding patterns obtained were analyzed with GelCompar II software (Applied Maths N.V., Sint-Martens-Latem, Belgium). The dice coefficient was used to calculate similarities between the banding patterns, and cluster analysis was conducted using the unweighted pair group method with arithmetic mean method to assess the clonal relationships of the isolates.

## 3. Results

**Figure 1:**
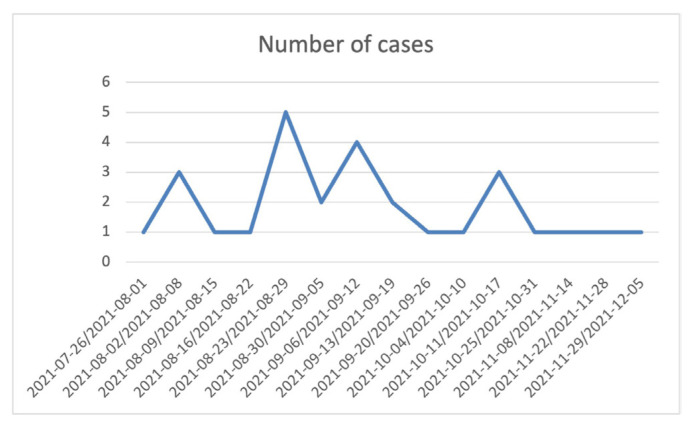
Curve of the outbreak.

**Figure 2:**
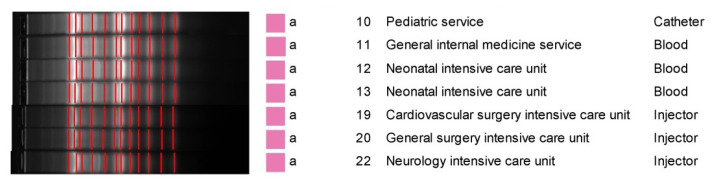
Results of the AP-PCR. The bacterial genotypes obtained from the samples of four patients (patients 1, 2, 3, and 4) were designated as genotypes 10, 11, 12, and 13, respectively. The bacterial genotypes obtained from syringe samples collected from various departments were designated as genotypes 19, 20, and 22 in this figure.

The first case of *R. insidiosa* was detected in the ICU on August 1st, 2021. *R. insidiosa* was isolated from the peripheral blood culture of that patient. In the same week, the second case of *R. insidiosa* was detected in the catheter blood culture of a different patient in the same ICU. *R. insidiosa* was detected in the peripheral and/or catheter blood cultures of 28 patients in adult and pediatric wards and ICUs until December 12th, 2021. Based on the outbreak analysis curve, a significant peak was observed in early September, with five cases in a single week, followed by smaller peaks mid-September and late October. This clustering pattern suggested a potential single-source outbreak or repeated exposure events (Figure 1). The Infection Control Committee of our university hospital evaluated the epidemiological data of the patients diagnosed with *R. insidiosa* bacteremia, including predisposing risk factors. No common risk factor was found. The mean age of these patients was 35 (range: 0–80) years. The patients were hospitalized in different wards. Standard procedures for outbreak analysis and healthcare-associated infections (HCAIs) were followed. To determine the source of the outbreak, tap water, saline, hand soap, antiseptic and antibiotic solutions, injectors, and arterial blood gas syringes were collected from the wards according to active surveillance procedures. All the samples were cultured, and *R. insidiosa* was isolated from arterial blood gas syringes with the same location and time-based identifier (LOT number). All the arterial blood gas syringes were recalled from the hospital departments and sent back to the manufacturer. The outbreak was reported to the national health authorities. Molecular epidemiological studies were conducted at the Department of Medical Microbiology, Faculty of Medicine, Turgut Özal University, located in Malatya. Isolated strains were sent to that center. Clonal analysis was performed using AP-PCR. A total of seven *R. insidiosa* isolates (four isolates from patients and three isolates from contaminated syringes) were included in this study. It was observed that the seven *R. insidiosa* isolates were monoclonal and identical (Figure 2). It was concluded that these contaminated arterial blood gas syringes caused the *R. insidiosa* bacteremia, especially in immunocompromised patients.

The results of the antibiotic susceptibility tests showed that all the isolates were sensitive to quinolones, imipenem, and piperacillin-tazobactam, but exhibited resistance to aminoglycosides. A total of 20 isolates were meropenem-resistant, and 12 of these isolates were ceftazidime-resistant (Table).

The outbreak was controlled by stopping the use of arterial blood gas syringes with the determined lot number. No new cases were detected within five months following the last case. Blood gas syringes with different lot numbers were used after ensuring no bacterial growth due to their practical use.

## 4. Discussion

Based on antibiotic susceptibility results and molecular epidemiological studies, a healthcare-associated bacteremia outbreak caused by *R. insidiosa* was detected. Contaminated arterial blood gas syringes were determined to be the common source of the outbreak. Prospective surveillance activities were conducted to find the source of the outbreak. An epidemiological investigation was initiated due to the repeated detection of a rare microorganism in the blood cultures of hospitalized patients. *Ralstonia* species can cause infections, especially in immunocompromised patients. Many studies presenting HCAIs and outbreaks caused by *Ralstonia* species can be found in the literature. The most common sources of contamination in these outbreaks were found to be intravenous drug and physiological saline solutions [9,10]. Contamination of these products generally occurs at the manufacturing stage. *Ralstonia* spp. can pass through 0.2-μm filters that are used to sterilize many medicinal products, such as saline solutions [11,12].

Generally, *R. pickettii* and *R. mannitolilytica* are more frequently detected as pathogens, whereas *R. insidiosa* is less common. Demirdag et al. [2], reported a similar outbreak of *R. pickettii* BSIs in pediatric leukemia patients, where contaminated saline solutions were identified as the source. Likewise, in the current study, contaminated arterial blood gas syringes were confirmed as the source of the *R. insidiosa*, demonstrating once again the vulnerability of sterile solutions and medical devices in hospital settings. Both studies emphasize the critical need for stringent infection control practices and heightened surveillance to quickly identify and address such contamination events. In another study, Ross et al. [13] reported an outbreak of BSIs caused by contaminated magnesium vials in ICUs. Shankar et al.[14] reported an outbreak of *R. mannitolilytica* in a hemodialysis unit, finding that sterile water used for intravenous drug preparations was contaminated by *Ralstonia*.

The outbreaks of *R. pickettii* revealed in recent reports from the UK and Germany emphasize the persistent threat caused by the contamination of medical products. The UK outbreak, associated with saline solutions, underscores the importance of robust surveillance and rapid response mechanisms to detect and mitigate such incidents. The genetic indistinguishability of isolates from patients in the UK indicates a common source of contamination, possibly in the manufacturing process of saline products. The subsequent recall and international cooperation were crucial in preventing further cases and ensuring patient safety [15]. Similarly, the German outbreak revealed *R. pickettii* in blood cultures, and genomic comparisons indicated a potential link to contaminated saline solutions. The genetic similarity of the isolates points to a single source of contamination, possibly in the supply chain of medical products. This is consistent with past data where outbreaks of *R. pickettii* were frequently associated with contaminated medical solutions, highlighting the bacterium’s ability to survive sterilization processes [16].

These two studies emphasize that low-virulence *R. pickettii* poses a significant risk to immunocompromised individuals. The findings highlight the need for strict quality control measures in the production of medical solutions and the importance of maintaining sterile conditions to prevent such contamination. Detecting and intervening in these outbreaks demonstrates the importance of implementing comprehensive infection control methods to manage and prevent HCAIs [15,16].

Jhung et al. [17] reported a national outbreak of *R. mannitolilytica* among pediatric patients in the US, which was associated with the use of a contaminated oxygen-delivery device. The investigation revealed intrinsic contamination of the device, emphasizing the importance of strict infection control and quality assurance measures when using medical devices. Despite manufacturer-recommended disinfection protocols, *Ralstonia* species were able to persist, leading to a recall of the device. This outbreak, much like others linked to *Ralstonia* contamination, underscores the potential for low-virulence pathogens to cause significant HCAIs, especially among vulnerable patient populations, and highlights the need for robust monitoring and prevention strategies in medical device usage.

## 5. Conclusion

The outbreak of *R. insidiosa* bacteremia associated with contaminated arterial blood gas syringes highlights the necessity for strict infection control protocols and rigorous quality assurance in the production of medical devices. The findings demonstrate the susceptibility of sterile products to contamination, posing significant risks to immunocompromised patients. This study underscores the critical importance of continuous surveillance and prompt interventions to prevent and manage HCAIs effectively.

## Figures and Tables

**Table:** Antibiogram susceptibility test results of isolates from the clonal analysis of 4 patients and the arterial blood gas syringes.

Antibiotic	Patient 1	Patient 2	Patient 3	Patient 4	Blood-gas syringe
Amikacin	R	R	R	R	R
Gentamicin	R	R	R	R	R
Imipenem	S	S	S	S	S
Levofloxacin	S	S	S	S	S
Meropenem	S	S	R	R	S
Piperacillin-tazobactam	S	S	S	S	S
Cefepime	S	S	S	S	S
Ceftazidime	S	S	R	S	S
Ciprofloxacin	S	S	S	S	S
Tobramycin	R	R	R	R	R

## Data Availability

The datasets analyzed during the study are available from the corresponding author on reasonable request.
